# Mitochondrial-related genes markers that predict survival in patients with head and neck squamous cell carcinoma affect immunomodulation through hypoxia, glycolysis, and angiogenesis pathways

**DOI:** 10.18632/aging.205081

**Published:** 2023-10-04

**Authors:** Zhonghua Li, Haoxi Cai, Jinyang Zheng, Xun Chen, Guancheng Liu, Yunxia Lv, Hui Ye, Gengming Cai

**Affiliations:** 1 Haicang Hospital Affiliated of Xiamen Medical College, Xiamen 361026, China; 2 Department of Otolaryngology Head and Neck Surgery, Quanzhou First Hospital Affiliated to Fujian Medical University, Quanzhou 362000, China; 3 School of Stomatology, Ningxia Medical University, Yinchuan 750004, China; 4 Department of Pathology, Quanzhou First Hospital Affiliated to Fujian Medical University, Quanzhou 362000, China; 5 Department of Oral Surgery, Quanzhou First Hospital Affiliated to Fujian Medical University, Quanzhou 362000, China; 6 Department of Otolaryngology Head and Neck Surgery, The Hospital Affiliated of Guilin Medical College, Guilin 541000, China; 7 Department of Thyroid Surgery, The Second Affiliated Hospital to Nanchang University, Nanchang 330006, China; 8 The School of Clinical Medicine, Fujian Medical University, Fuzhou 361026, China; 9 The Graduate School of Fujian Medical University, Fuzhou 361026, China

**Keywords:** mitochondrial-related genes, head and neck squamous cell carcinoma, immunomodulation, risk score, prognostic model, predicting survival

## Abstract

Mitochondria play a crucial role in the occurrence and development of tumors. We used mitochondria-related genes for consistent clustering to identify three stable molecular subtypes of head and neck squamous cell carcinoma (HNSCC) with different prognoses, mutations, and immune characteristics. Significant differences were observed in clinical characteristics, immune microenvironment, immune cell infiltration, and immune cell scores. TP53 was the most significantly mutated; cell cycle-related pathways and tumorigenesis-related pathways were activated in different subtypes. Risk modeling was conducted using a multifactor stepwise regression method, and nine genes were identified as mitochondria-related genes affecting prognosis (DKK1, EFNB2, ITGA5, AREG, EPHX3, CHGB, P4HA1, CCND1, and JCHAIN). Risk score calculations revealed significant differences in prognosis, immune cell scores, immune cell infiltration, and responses to conventional chemotherapy drugs. Glycolysis, angiogenesis, hypoxia, and tumor-related pathways were positively correlated with the RiskScore. Clinical samples were subjected to qPCR to validate the results. In this work, we constructed a prognostic model based on the mitochondrial correlation score, which well reflects the risk and positive factors for the prognosis of patients with HNSCC. This model can be used to guide individualized adjuvant and immunotherapy in patients with HNSCC.

## INTRODUCTION

Predominantly, head and neck squamous cell carcinoma (HNSCC) manifests as the primary histological variant of head and neck cancers, emerging as the world’s sixth most ubiquitous malignancy, encompassing over 90% of cases. Factors such as genetic predispositions, interaction with tobacco carcinogens, immoderate alcohol indulgence, and HPV infections are acknowledged determinants for HNSCC. Regrettably, the vast majority of HNSCC cases are discerned in their advanced phases. Even with significant progress in diagnostic and therapeutic strategies, the quintessential five-year survival trajectory remains unyielding at 50%. Consequently, the pressing imperative is to unearth steadfast molecular markers to optimize HNSCC clinical interventions [1].

Mitochondria are organelles with notable, metabolic activities and are considered signaling hubs with biosynthetic, bioenergetic, and signaling functions responsible for coordinating key biological pathways [2]. Mitochondria are associated with various diseases, namely cardiovascular, neurological, and metabolic disorders. Furthermore, mitochondria can influence all the processes involved in tumor formation and progression. As a result, they can affect all tumorigenic processes by regulating the metabolic, oxidative, and apoptotic processes in cancer cells. Mitochondrial DNA defects in mitochondrial functional defects have been reported in several cancers. Beyond their pivotal bioenergetic roles, mitochondria underpin tumor anabolism, orchestrate redox and calcium equilibrium, guide transcriptional governance, and regulate cellular demise. Moreover, mitochondrial dynamics hold significance in stress signaling. Cancer formation and progression are closely related to mitochondria; however, much regarding this complex relationship remains unclear [3, 4].

In this research, we harnessed mitochondria-associated genes to delineate enduring molecular subclasses through uniform clustering, juxtaposing the clinical, pathway, and immune attributes across these subcategories. Vital prognostic influencers rooted in mitochondria related genes were discerned via multi-factorial stepwise regression, culminating in the formulation of a clinical risk paradigm for such genes. To enhance the prognostic framework and fortify survival forecasts, we amalgamated the RiskScore with clinicopathological nuances, crafting a nomogram to appraise the peril faced by HNC patients, thereby paving the way for tailored therapeutic approaches.

## RESULTS

### Establishment of molecular subtypes

**Figure 1:**
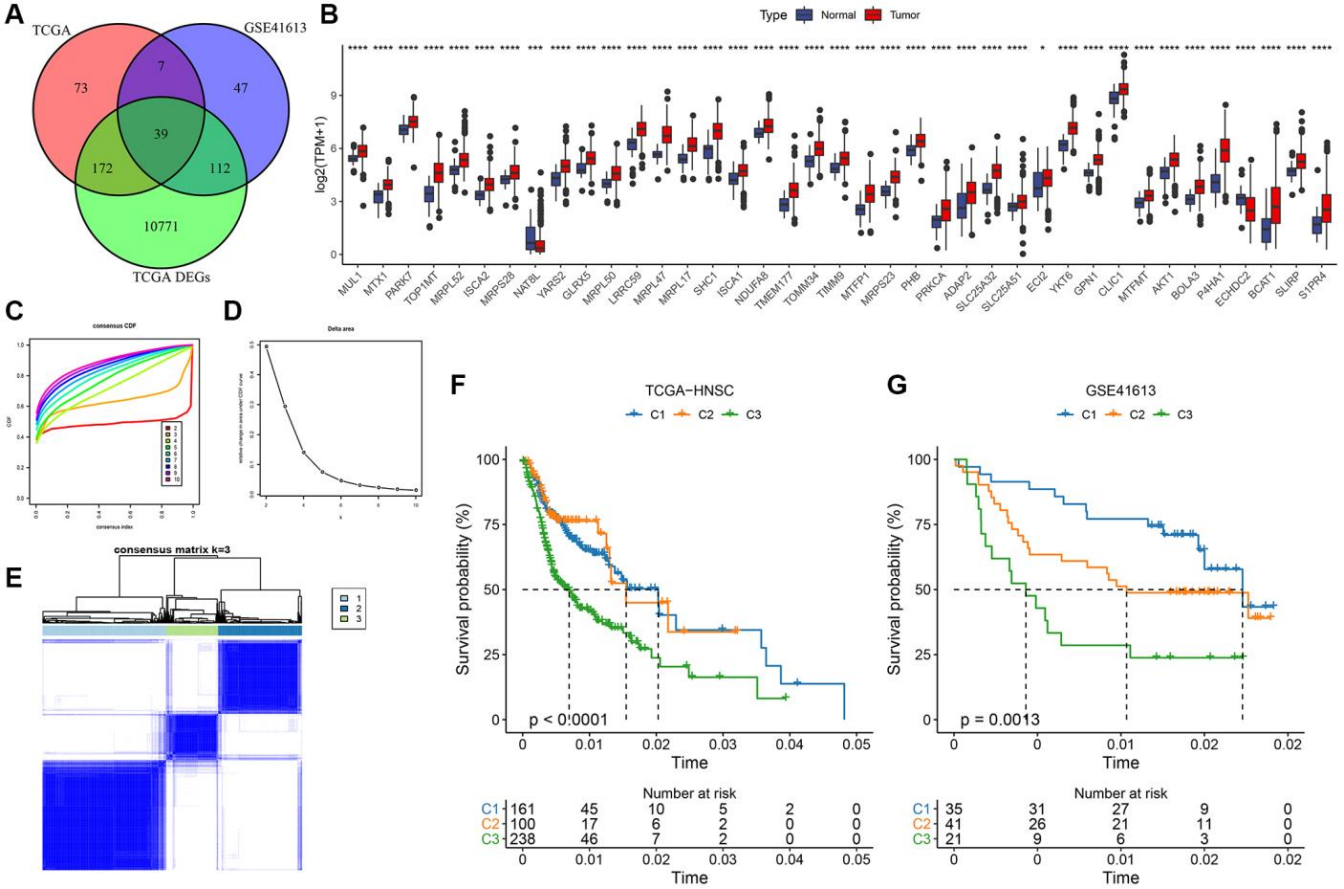
**Molecular typing based on mitochondrial-related genes.** (**A**) Identification of differential mitochondria-related genes with significant prognosis; (**B**) Expression of 39 genes in tumor and paraneoplastic normal samples; (**C**) CDF curves of TCGA cohort samples; (**D**) CDF Delta area curves of TCGA cohort samples, Delta area curve of consensus clustering, indicating the relative change in area under the cumulative distribution function (CDF) curve for each category number k compared with k – 1. The horizontal axis represents the category number k, and the vertical axis represents the relative change in area under the CDF curve; (**E**) Heat map of sample clustering at consensus k = 3; (**F**) KM curves of the relationship among the prognosis of the three subtypes of TCGA; (**G**) KM curves of the prognosis of the three subtypes in the GSE41613 cohort.

Differential analysis was performed using the limma package on tumor and normal samples next to cancer in TCGA (|FC|>1.2 and FDR <0.05, Supplementary Table 1). Next, a one-way Cox analysis of mitochondria-related genes was performed in GSE41613 in TCGA-HNSC (*P* < 0.05), and the differential genes were intersected with mitochondrial genes with a significant prognosis in both datasets. The final results included 39 mitochondria-related differentially expressed genes with a significant prognosis (Figure 1A). Tumor samples expressed most of these genes (Figure 1B). Patients were then classified based on the coherent grouping of expression patterns connected to 39 key mitochondria-related genes with significant prognostic implications. The ideal cluster count was determined using the cumulative distribution function (CDF), and the CDF Delta area curve showed that selecting three clusters increased stability (Figure 1C, 1D). This final choice of k = 3 culminated in three distinct molecular subtypes: C1, C2, and C3 (Figure 1E). Delving deeper into the prognostic attributes of these subtypes revealed marked disparities in their prognostic outcomes. As depicted in Figure 1F, C1 boasted the most favorable prognosis, succeeded by C2, while C3 manifested the least favorable outcome. Employing an analogous methodology for the GSE41613 dataset, these molecular subtypes again exhibited pronounced prognostic variations (e.g., Figure 1G), resonating with the findings from the TCGA dataset (Supplementary Tables 2 and 3 detail the clustering outcomes for TCGA and GSE41613 subtypes).

### Clinical characteristics among subtypes

**Figure 2:**
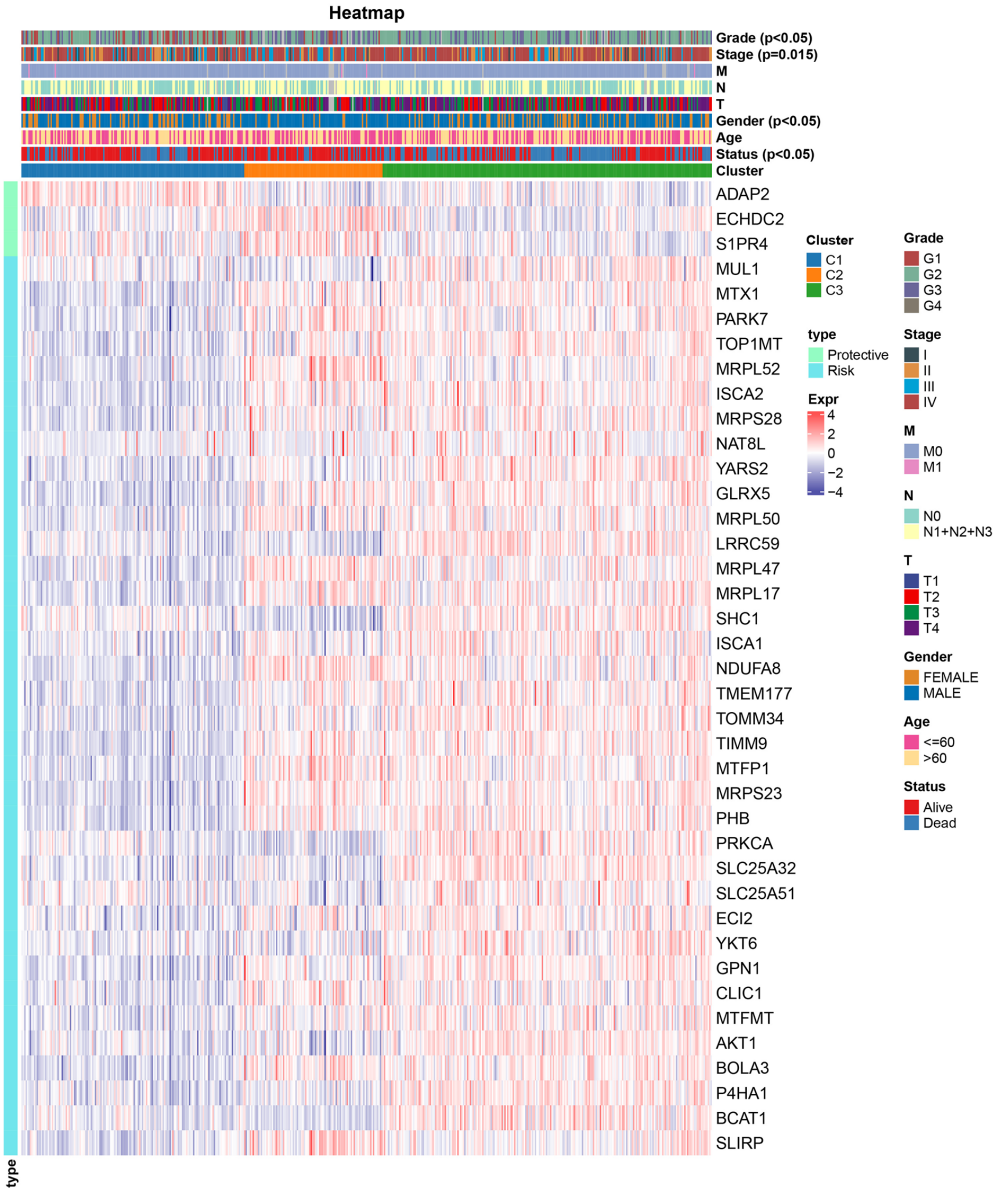
Relationship between gene expression profiles and clinical characteristics among molecular subtypes.

We assessed the clinicopathological attributes across TCGA-HNSC molecular subtypes, uncovering pronounced distinctions in gender, clinical progression, staging, gradation, and survival outcomes among the three categories (Figure 2). Furthermore, in examining the expression of 39 mitochondria-related genes bearing prognostic relevance across distinct molecular subtypes, we discerned that the “Risk” gene was preeminent in C3, whereas the “Protective” gene exhibited pronounced elevation in C1.

### Inter-subtype immune profile

**Figure 3:**
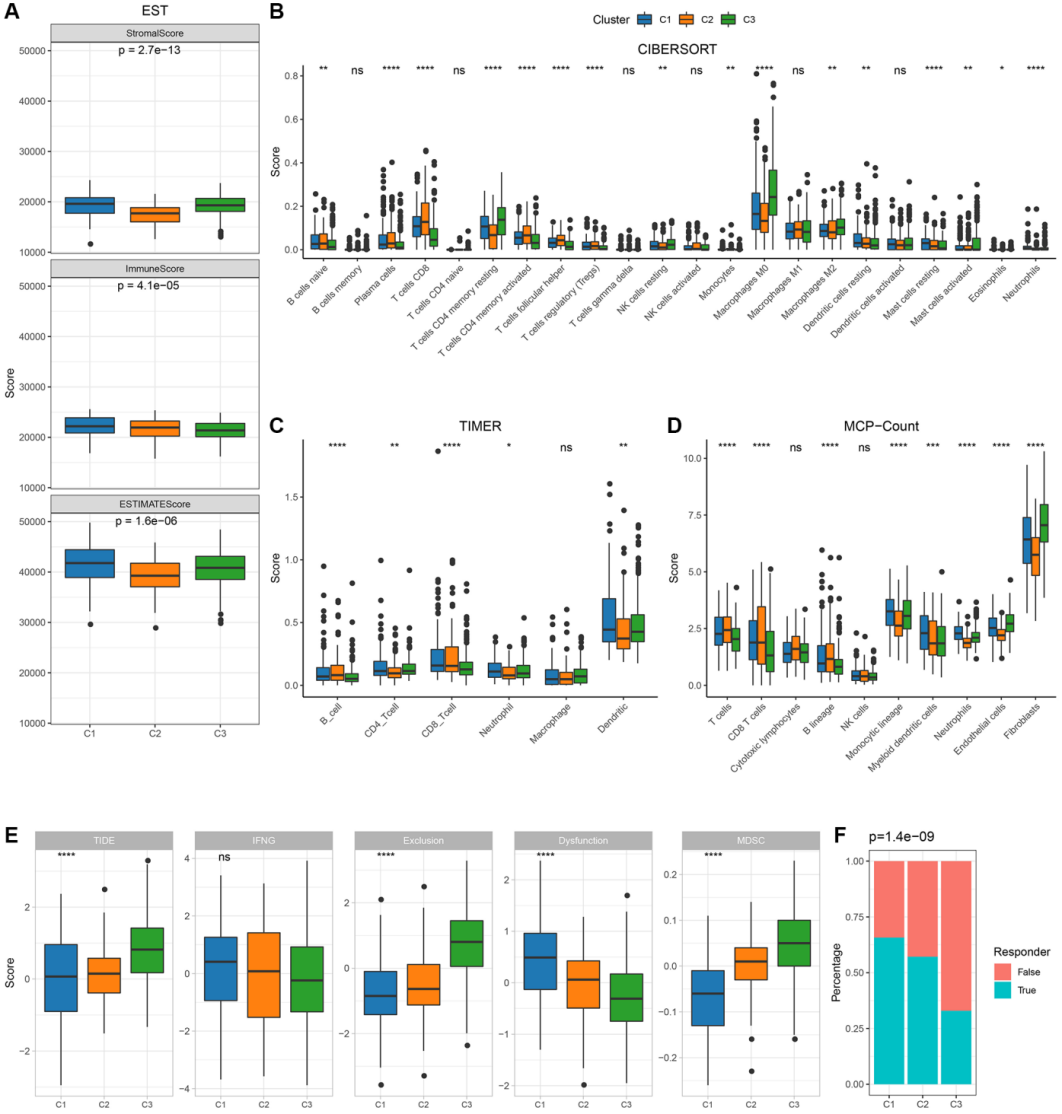
**Immunological characteristics between molecular subtypes in TCGA cohort.** (**A**) Differences in TCGA cohort ESTIMATE immune scores between molecular subtypes; (**B**) Differences in TCGA cohort TIDE scores and immune response between molecular subtypes CIBERSORT immune scores between molecular subtypes; (**C**) Differences in TCGA cohort TIMER calculated immune cell scores between molecular subtypes; (**D**) Differences in TCGA cohort MCP-Count immune scores between molecular subtypes; (**E**, **F**): TCGA cohort TIDE scores and immune response between molecular subtypes.

To delineate the immune milieu across varied molecular subtypes, we scrutinized immune cell penetration employing ESTIMATE, revealing a markedly diminished “ImmuneScore” for C3 compared to its counterparts (Figure 3A), characterized by attenuated immune engagement. Utilizing the CIBERSORT methodology, we approximated the distribution of immune cell variants, identifying disparities among the molecular classifications (Figure 3B). Immune engagement evaluations were further determined via MCP-count and TIIMER, with a majority of immune cell metrics manifesting significant variations across subtypes (Figure 3C, 3D). Subsequently, the TIDE software provided insights into the prospective therapeutic implications of immunotherapy for the identified molecular categories. Elevated TIDE prognostications signify an increased propensity for immune evasion, hinting at a diminished therapeutic responsiveness to immunotherapy. Evidently, as depicted in Figure 3E, C3 from the TCGA cohort, marked by an adverse prognosis, displayed a considerably amplified TIDE metric, insinuating an escalated tendency for immune evasion and diminished therapeutic prospects from immunotherapy relative to the alternate subtypes. This is further underscored by the minimalistic response rates to immune checkpoint inhibitors in C3 (Figure 3F).

### Inter-subtype mutation characterization/pathway characterization

**Figure 4:**
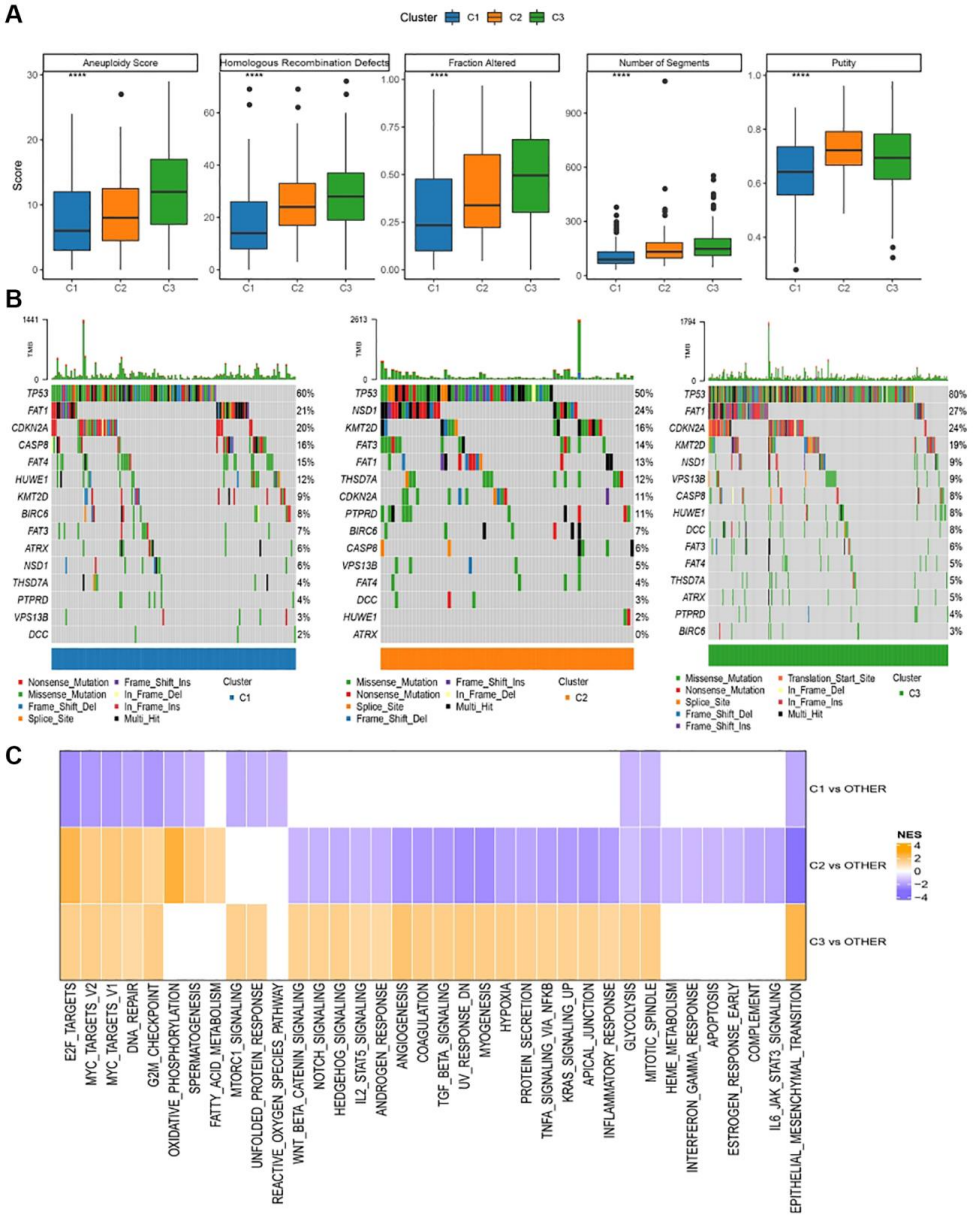
**Genomic alterations in molecular subtypes of TCGA cohort.** (**A**) Comparison of homologous recombination defects, aneuploidy score, fraction altered, number of segments, tumor purity in molecular subtypes of TCGA cohort differences; (**B**) Somatic mutations in the three molecular subtypes; (**C**) GSEA results among molecular subtypes of TCGA cohort.

Disparities in genomic modifications between these two molecular subtypes within the TCGA cohort underwent meticulous analysis. Insights into the molecular attributes of TCGA-HNSCs were extrapolated from referenced literature [5, 6]. C3 exhibited an elevated aneuploidy index, proportion altered, segment count, surpassing both C1 and C2. Conversely, C1 demonstrated notably diminished tumor purity relative to C2 and C3 (Figure 4A). Subsequently, the mutation dataset, refined by TCGA mutect2 software, was acquired. Genes surpassing a mutation frequency of three were shortlisted for salient high-frequency mutations within each subtype via the Fisher test, adhering to a selection benchmark of *P* < 0.05. The mutational idiosyncrasies of the paramount 15 genes within each subtype are delineated in in Figure 4B. Subsequently, the manifestation of variably activated pathways across distinct molecular subtypes was scrutinized. For pathway identification, we executed a GSEA, leveraging the h.all.v7.5.1.entrez.gmt gene set from the MSigDB database [7], with FDR <0.05 denoting noteworthy enrichment. Figure 4C portrays the outcomes of the TCGA assessment, revealing an inhibition of cell cycle-associated pathways in C1, juxtaposed with the activation of both cell cycle and tumorigenesis-associated pathways.

### Identification of differential genes

**Figure 5:**
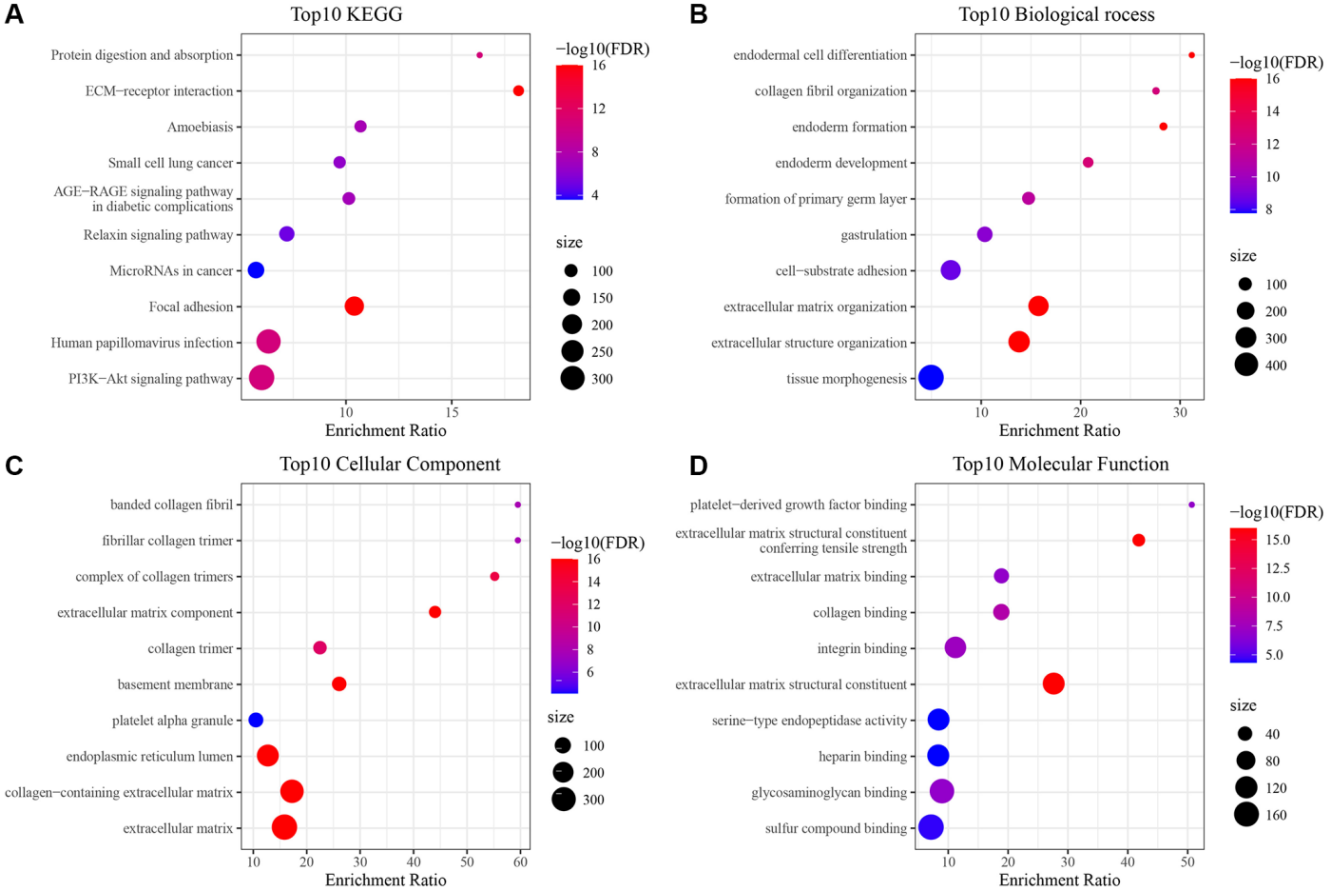
**Enrichment results of GO and KEGG pathways of differentially upregulated genes in C3 subtype.** (**A**) Results of KEGG analysis of upregulated genes in TCGA-HNSC cohort C3; (**B**–**D**) Results of GO analysis of upregulated genes in TCGA-HNSC cohort C3.

In our prior analysis in this paper, three molecular subtypes were established based on prognostically significant differential mitochondria-associated genes that differed in clinical, immune, and pathway features. Subsequently, genes exhibiting differential expression amongst C1, C2, and C3 relative to other subtypes were discerned utilizing the limma package, adhering to criteria of FDR <0.05 and |log2FC| > 1. This analysis culminated in the identification of 224, 443, and 226 distinctively expressed genes in C1, C2, and C3, respectively, aggregating to an ultimate count of 662 unique genes (Supplementary Tables 4–6). Additionally, a comprehensive functional enrichment assessment of these differentially expressed genes was undertaken via the R software’s clusterProfiler package (FDR <0.05). The enriched findings from both GO and KEGG pathways pertinent to genes predominantly elevated in C3 isoforms are depicted in Figure 5 (Supplementary Table 7).

### Risk modeling

**Figure 6:**
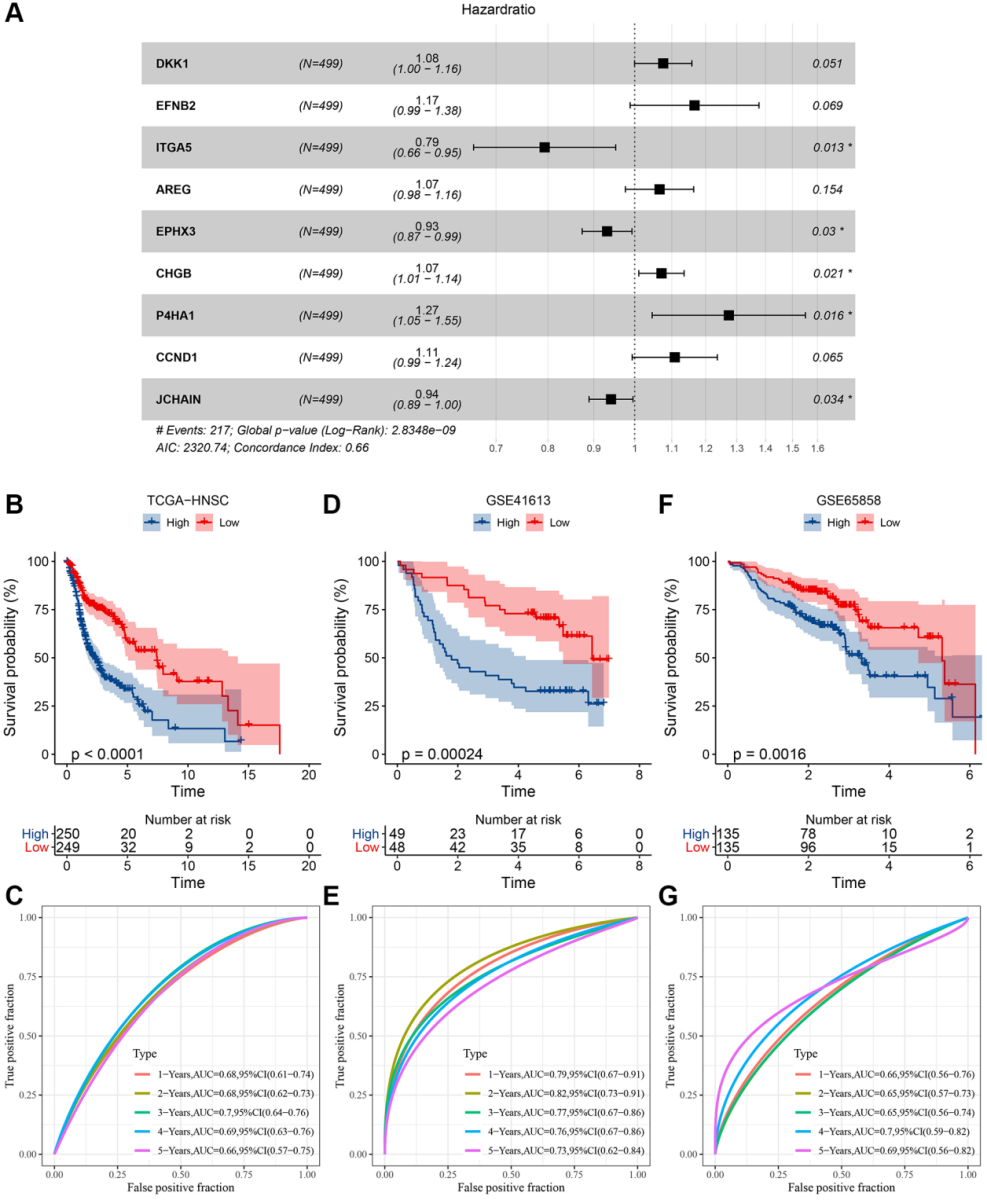
**Determination of risk model and its KM, ROC curves.** (**A**) Multifactor forest plot of prognostic key genes; (**B**) KM curve of risk model constructed for 9 genes in TCGA dataset; (**C**) ROC curve of the risk model in TCGA dataset; (**D**) KM curve of risk model constructed for 9 genes in GSE41613 dataset; (**E**) ROC curve of risk model in GSE41613 dataset; (**F**) KM curve of risk model constructed for 9 genes in GSE65858 dataset; (**G**) ROC curve of risk model in GSE65858 dataset.

Utilizing the coxph function from the SURVIVAL package, a univariate Cox analysis was conducted on the 662 differential genes, pinpointing 20 genes of profound prognostic significance (*P* < 0.001) as illustrated in Supplementary Table 8. Subsequent to this, a multifactorial stepwise regression analysis was undertaken. This analytical approach employs the AIC information criterion—a metric balancing the statistical fit against the parameter count in the model. Using the AIC method within the MASS package, the analysis commences with the most intricate model, systematically excluding variables to minimize the AIC value. A diminished value signifies an optimal model, suggesting a satisfactory fit achieved with minimal parameters. Ultimately, nine pivotal genes influencing prognosis were discerned, as exemplified in Figure 6A. The definitive model equation is presented below: RiskScore = 0.073 × DKK1 + 0.154 × EFNB2 + (−0.232 × ITGA5) + 0.064 × AREG + (−0.071 × EPHX3) + 0.069 × CHGB + 0.242 × P4HA1 + 0.103 × CCND1 + (−0.061 × JCHAIN) Subsequently, risk scores for each specimen within the TCGA cohort were derived utilizing the expression metrics of the nine distinct genes. Based on the median RiskScore, TCGA samples were segregated into high-risk and low-risk contingents, and the Kaplan-Meier curves demonstrated a pronounced disparity between these cohorts (Figure 6B). The timeROC R package facilitated an ROC analysis on the prognostic stratification by RiskScore, examining the predictive efficacy over intervals from 1 to 5 years. The model exhibited an impressive area beneath the AUC curve (Figure 6C). In an endeavor to reinforce the model’s robustness validation, analogous methodology was applied to authenticate the GSE41613 and GSE65858 datasets. These validations confirmed a commendable AUC area, with conspicuous contrasts between high-risk and low-risk classifications (Figure 6D–6G).

### Nomogram

**Figure 7:**
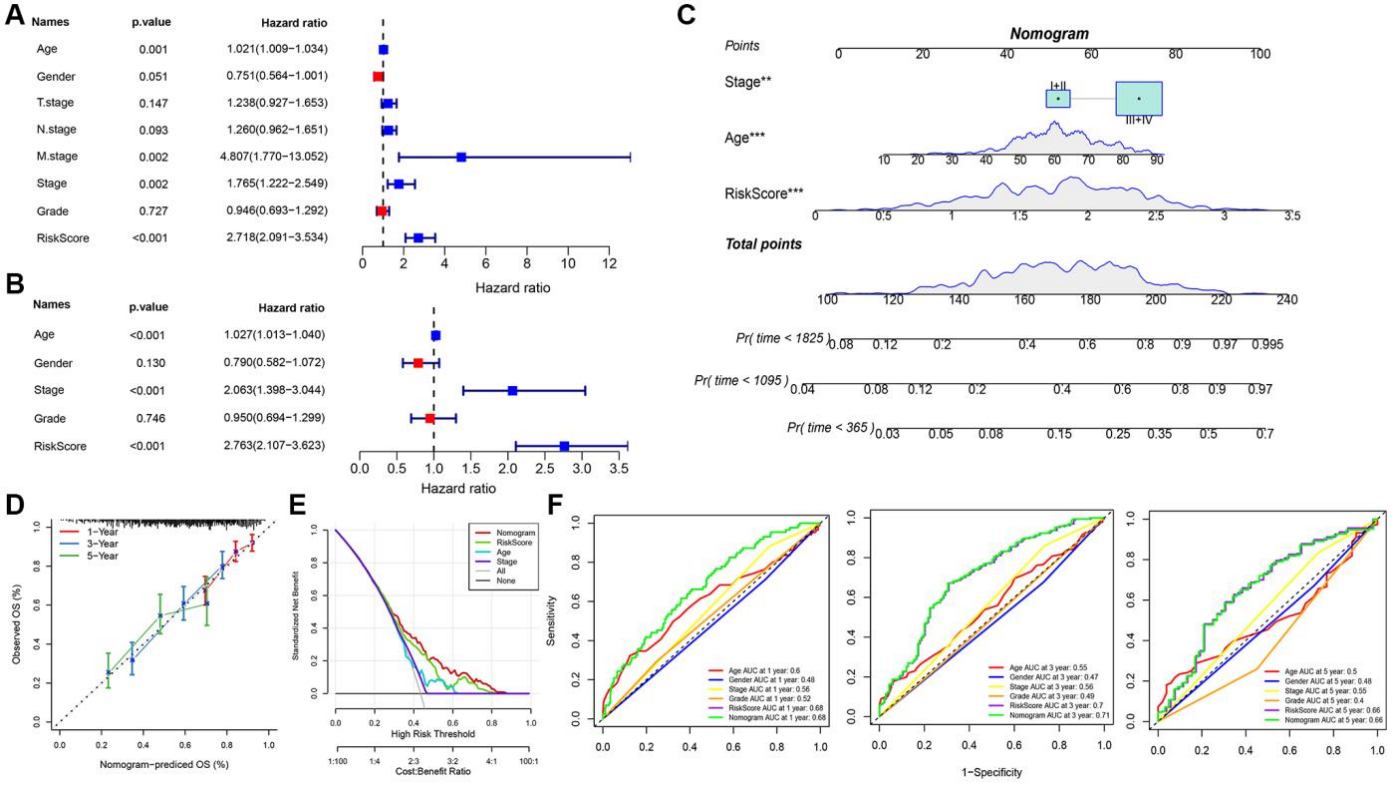
**Determination and survival prediction ability of nomogram.** (**A**) Single-factor forest plot of RiskScore and clinical features; (**B**) Multifactor forest plot of RiskScore and clinical features; (**C**) RiskScore combined with clinical features column line plot; (**D**) Calibration curves of column line plot at 1, 3, and 5 years; (**E**) Decision curve of column line plot; (**F**) ROC curves of various clinical features for overall survival (OS) at 1, 3, and 5 years.

**Figure 8:**
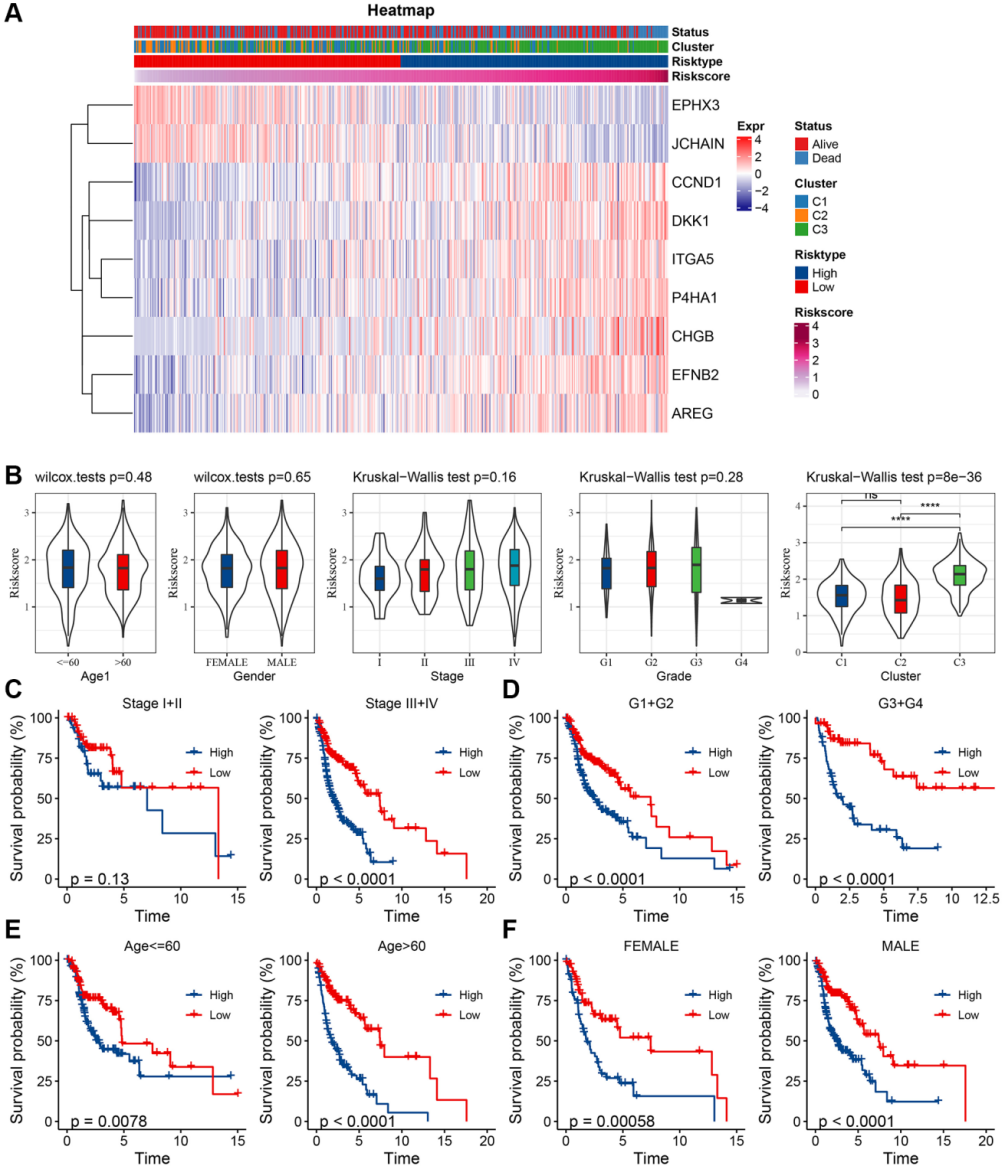
**Clinical characteristics between risk groups.** (**A**) Key gene expression in relation to clinical characteristics and RiskScore; (**B**) Differences between RiskScore between clinicopathological subgroups in TCGA-HNSC cohort; (**C**) KM curves between RiskScore of the high- and low-risk groups for different stages in TCGA-HNSC cohort; (**D**) KM curves between RiskScore high- and low-risk groups for grades in TCGA-HNSC cohort grade; (**E**) KM curves between RiskScore high- and low-risk groups of age groups in TCGA-HNSC cohort; (**F**) KM curves between the RiskScore of high- and low-risk of different genders in TCGA-HNSC cohort.

Univariate and multifactorial Cox regression assessments of RiskScore, juxtaposed with clinical attributes, ascertained that RiskScore, age, and stage stood as paramount prognostic determinants (Figure 7A, 7B). To crystallize the interplay between risk evaluation and patient survival trajectories, we melded the RiskScore with pertinent clinicopathological indicators, giving rise to an illustrative columnar depiction (Figure 7C); here, RiskScore emerged as the preeminent force in forecasting survival. The model’s prognostic fidelity underwent scrutiny via the calibration trajectory depicted in Figure 7D. Calibration curves at the 1-, 3-, and 5-year milestones were in near-congruence with the benchmark trajectory, underscoring the model’s adept prognostic prowess. The model’s robustness garnered further validation through DCA (Decisioncurve); wherein both RiskScore and the nomogram exhibited a palpable ascendancy over extreme curves (Figure 7E). As illuminated in Figure 7F, the ROC curve analysis elucidated that the nomogram, coupled with RiskScore, manifested unparalleled sensitivity and specificity in forecasting the overarching survival of head and neck carcinoma patients, eclipsing other pertinent clinical markers such as age, gender, histological caliber, and TNM clinical echelon, thus asserting their superlative prognostic merit. To elucidate the nexus between the RiskScore and TCGA-HNSC clinical attributes, we probed the variances in risk stratifications and scores vis-à-vis clinical gradations utilizing the TCGA-HNSC compendium. The representation of C3 preponderated within the high-risk cohort (Figure 8A), whilst the RiskScore ascended concomitantly with the clinical tier (Figure 8B). Additionally, juxtapositions of the RiskScore across molecular subtypes revealed C3, bearing the most ominous prognosis, also brandished the zenith RiskScore. We further juxtaposed the prognostic disparities between high and low RiskScore strata across diverse clinicopathological categorizations. The results demonstrated that the risk groupings were equally good in different clinical groupings, proving the reliability of the risk groupings (Figure 8C–8F).

### Immune/pathway characteristics among risk subgroups

**Figure 9:**
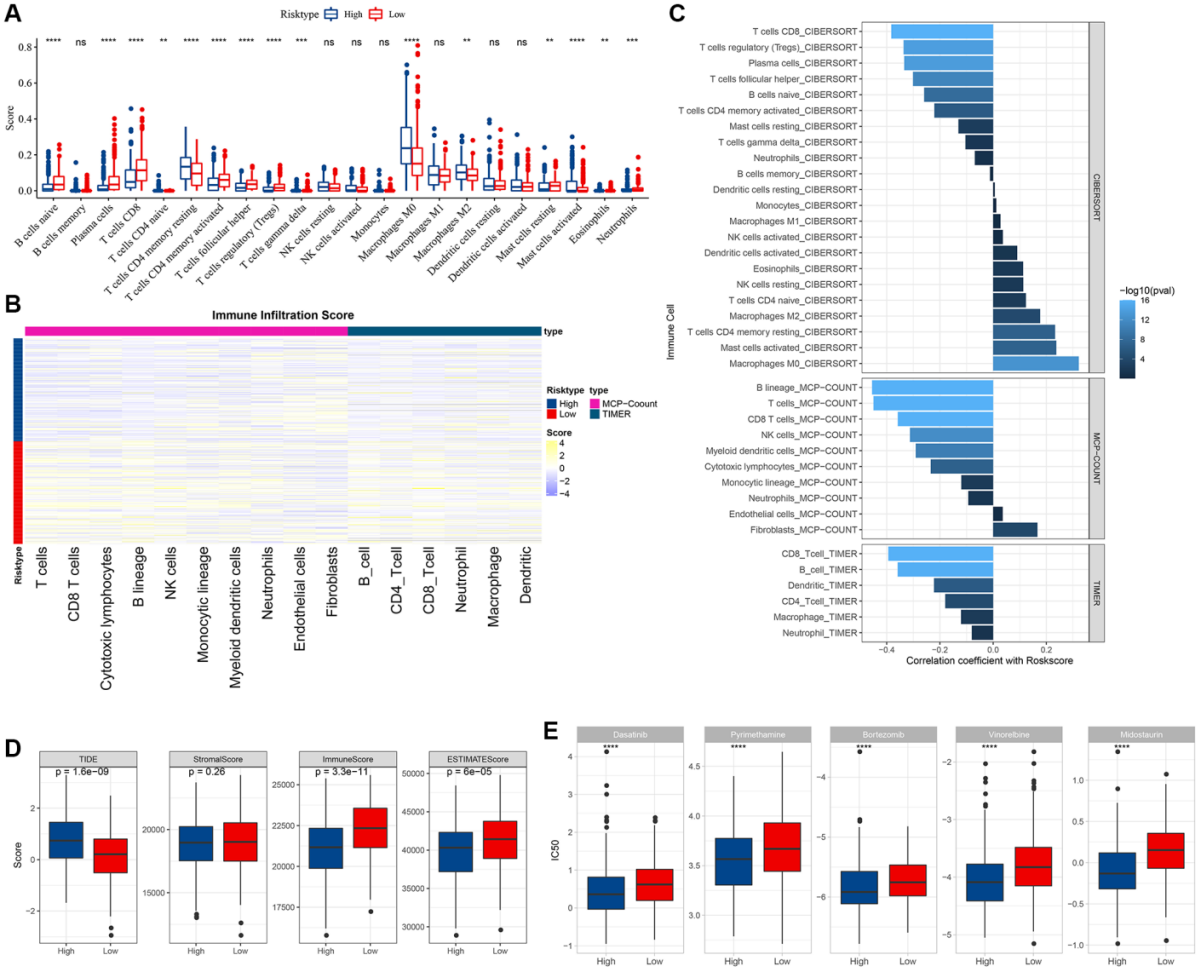
**Immune and pathway characteristics between different risk groups.** (**A**) difference in CIBERSORT immune infiltration between risk subgroups in TCGA cohort; (**B**) difference in MCP-count, TIMER immune score between risk subgroups in TCGA cohort; (**C**) correlation between immune score and RiskScore in TCGA cohort; (**D**) difference in TIDE score and ESTIMATE immune score between risk subgroups in TCGA cohort; (**E**) difference in drug sensitivity (IC50) between risk subgroups in TCGA cohort.

**Figure 10:**
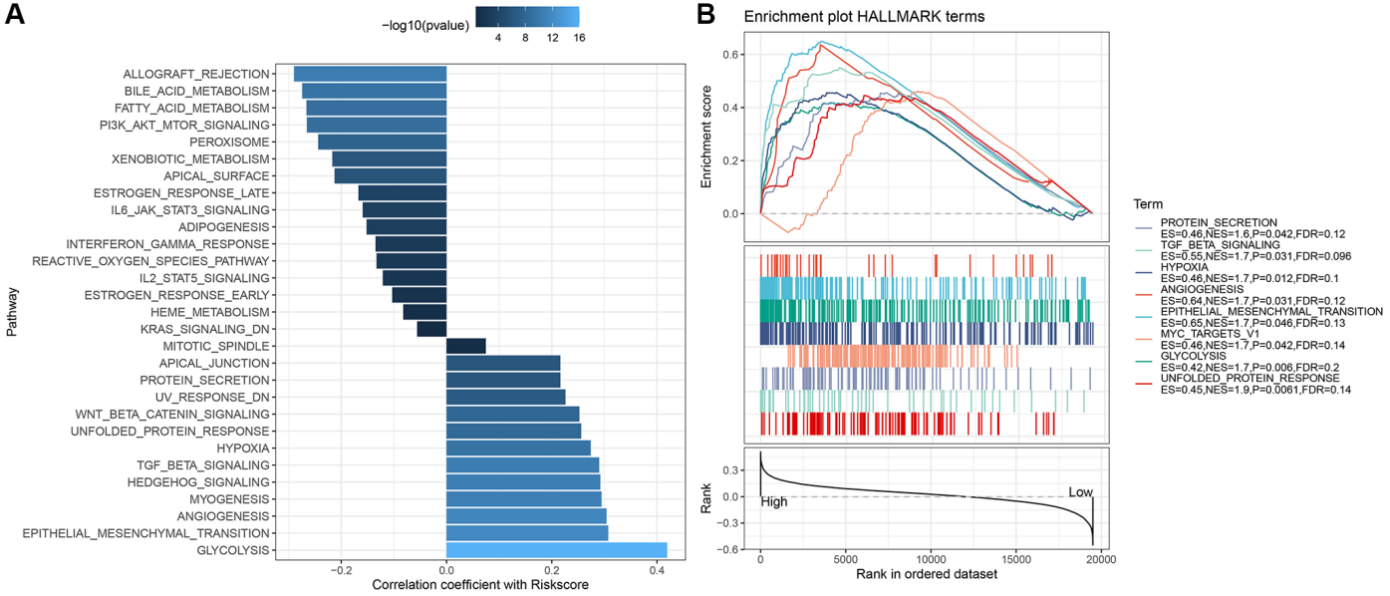
**Correlation between differential pathways and RiskScore in risk groups and their GSEA results in TCGA dataset.** (**A**) Correlation between risk intergroup differential pathways and RiskScore in TCGA dataset; (**B**) GSEA results for high- and low-risk groups in TCGA dataset.

To discern variances in the immune microenvironment across risk strata, we employed the CIBERSORT algorithm to quantify immune cell prevalence within TCGA-HNSC’s high- and low-risk factions (Figure 9A). Utilizing both MCP-count and TIMER methodologies, we derived the immune infiltration indices for the TCGA-HNSC cohort, revealing pronounced disparities in most immune cell indices between risk strata (Figure 9B). The “ImmuneScore” was markedly elevated in the low-risk segment compared to its high-risk counterpart. Analyzing immunotherapeutic divergences between risk sectors within the TCGA cohort, we harnessed the TIDE software to gauge potential clinical immunotherapy outcomes for both risk sectors. Notably, the TIDE score for the high-risk faction surpassed that of the low-risk, implying an augmented propensity for immune evasion in the former, yet paradoxically suggesting a greater therapeutic benefit potential. Conversely, the amplified TIDE score in the high-risk cohort indicated increased susceptibility to immune subterfuge and a diminished probability of immunotherapeutic success (Figure 9D). We also employed the Pearson approach to deduce the correlation between RiskScore and immune score, unearthing a pronounced inverse association between RiskScore and a majority of immune cell indices (Figure 9C). Furthermore, we assessed the responsiveness to conventional chemotherapeutics across TCGA cohort subgroups, observing heightened sensitivity within the high-risk cohort compared to its low-risk counterpart (Figure 9E). To delve deeper into the nexus between RiskScore and biological functions across diverse samples, we employed the TCGA dataset, designating h.all.v7.5.1.symbols.gmt as our gene set. Thereafter, we executed single-sample GSEA (ssGSEA) analyses via the R package GSVA. For each distinct function, the ssGSEA enrichment scores were derived for every sample. Employing the rank sum test, we discerned distinct pathways between risk groups (*P* < 0.05) and further evaluated the interrelation between these pathways and the RiskScore. As illustrated in Figure 10A, pathways integral to glycolysis, angiogenesis, hypoxia, and specific tumorigenic processes manifested a robust positive association with the RiskScore. Through GSEA, we also analyzed prominently enriched pathways within both high- and low-risk cohorts. Adopting a threshold of NP <0.05, we pinpointed the saliently enriched pathways (Figure 10B).

### Expression of mitochondrial prognostic-related genes in HNSCC

**Figure 11:**
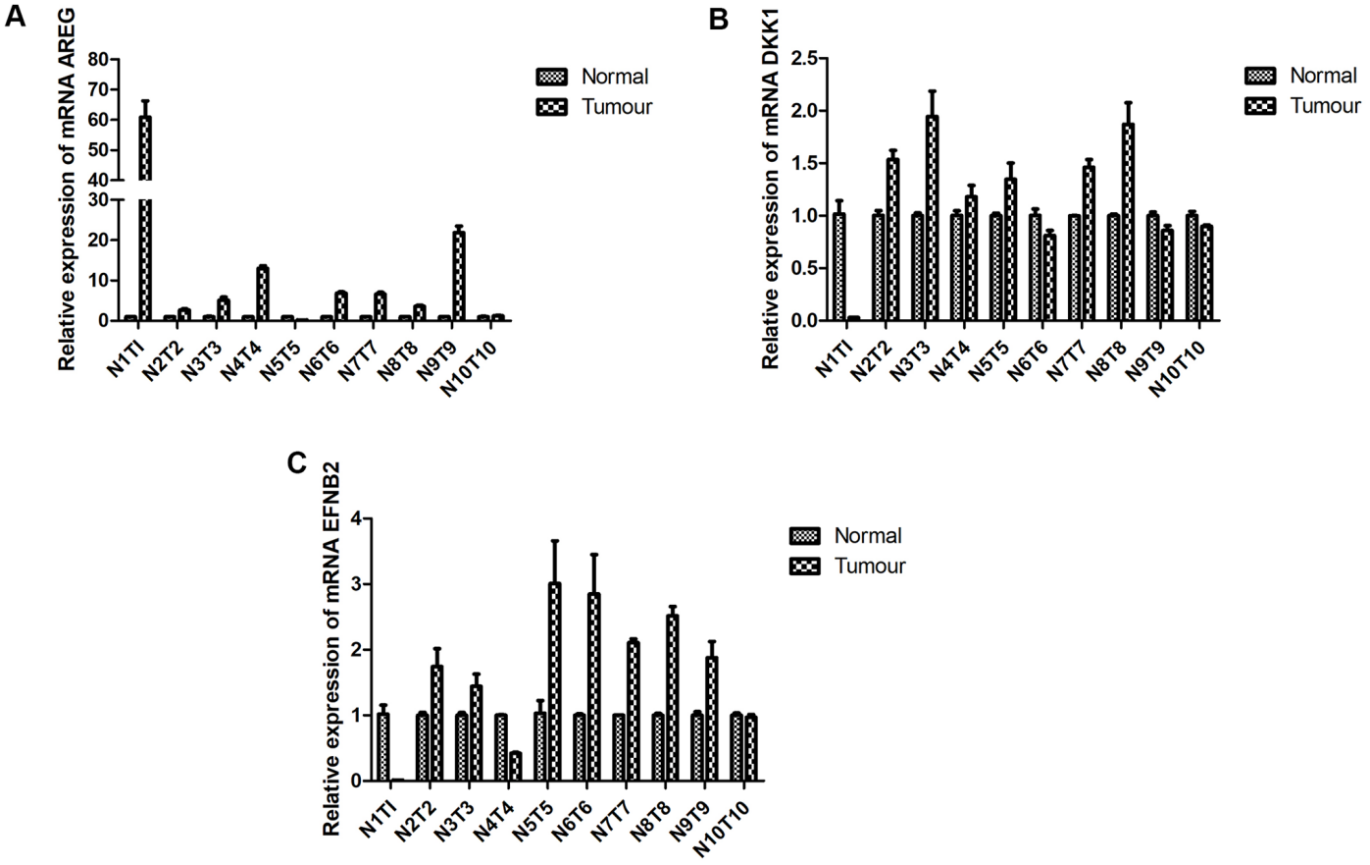
**RT-qPCR. Expression of prognostic-specific genes in HNSCC were consistent with the predicted trend.** RT-qPCR detected mRNA expression levels of (**A**) AREG, (**B**) DKK1, and (**C**) EFNB2 in the tumor tissue and adjacent tissue of patients with HNSCC *p* < 0.05.

Prior findings indicated that CCL22, CTSG, and FGD3 expressions were auspiciously linked with HNSCC prognosis, while TPP1 exhibited an inverse correlation. Using RT-qPCR, we probed the mRNA expression levels of these four genes in both tumor and adjacent non-tumorous tissues from 10 HNSCC patients. Notably, expressions of (A) AREG (observed in 8 of 10 samples, or 80%; Figure 11A), (B) DKK1 (observed in 6 of 10 samples, or 60%; Figure 11B), and (C) EFNB2 (observed in 7 of 10 samples, or 70%; Figure 11C) in tumorous specimens were markedly elevated compared to their non-tumorous counterparts. These data underscore that the expression patterns of certain prognostic genes in HNSCC align seamlessly with our anticipations.

## DISCUSSION

While aberrations in mitochondrial genes frequently manifest in cancer cells, they seldom incapacitate mitochondrial energy metabolism. Instead, they recalibrate the mitochondria’s bioenergetic and biosynthetic equilibrium. Such altered states liaise with the nucleus via ‘retrograde signaling’, orchestrating a gamut of signal transduction pathways, transcriptional networks, and chromatin configurations, adeptly catering to the mitochondrial and nuclear exigencies of malignancies. Subsequently, malignant cells reconfigure neighboring stromal cells, refining the tumoral milieu. Mitochondria, pivotal in tumor anabolism, are custodians of redox balance, calcium homeostasis, transcriptional oversight, and cellular demise. Tumorigenesis, progression, and therapeutic responses are intricately intertwined with host immunological processes, many of which pivot on unimpaired mitochondrial metabolism [2, 4]. Reactive oxygen species (ROS) don pivotal roles—spanning cell death regulation, DNA repair, stem cell sustenance, metabolic shifts, and sculpting the tumor microenvironment. Intriguingly, such ROS also influence T-cell dynamics. In HNC, modulating ROS levels during chemotherapy/radiotherapy is of paramount clinical import. An array of non-oncologic pharmaceuticals, molecular compounds, traditional herbal remedies, and avant-garde interdisciplinary methodologies—encompassing photodynamics, nanotech systems, and Bio Electro-Magnetic-Energy-Regulation (BEMER) therapies—can adeptly modulate cellular ROS in HNC, potentiating the efficacy of conventional treatment modalities [8–11]. Rencelj reported posited that mitochondrial receptors incisively target pivotal metabolic entities, influencing myriad oncogenic signaling cascades and holding sway over the Warburg effect—a phenomenon vital for cancer cell proliferation. A nexus between mitochondria and hypoxia has been delineated in breast cancer and HNCs [12]. Lee et al. elucidated that the majority of HNCs, rooted in mucosal epithelial cells, retain epithelial characteristics. However, as malignancy advances, it often morphs into mesenchymal or hypofractionated phenotypes, paving the way for invasion, metastasis, and treatment resistance. Erosion of epithelial traits, courtesy of epithelial-mesenchymal transition, might predispose such tenacious malignancies to ferroptosis. Steering mitochondrial or iron metabolism can amplify intracellular ferrous iron and lipid peroxidation, rendering drug-resistant neoplasms more amenable to ferroptosis [13]. Huang and associates postulated that metabolic shifts originating from nasopharyngeal carcinoma cells might foster tumor advancement and immunosuppression via intercellular discourse with adjacent immune cells. Crafting therapeutic targets at this metabolic-immune interface could herald novel treatment avenues for nasopharyngeal carcinoma [14]. Ergo, strategic manipulation of mitochondrial metabolism emerges as a promising therapeutic frontier for HNC. In our investigation, we discerned a differential expression of mitochondrial-associated genes between tumoral and adjacent healthy tissues, pinpointed molecular isoforms through robust clustering, and spotlighted nine pivotal mitochondrial-associated genes. Among them, CCND1, located on chromosome band 11q13, codes for cell cycle protein D1, pivotal in orchestrating G1-S phase transition. Beyond its pro-proliferative prowess, it augments cellular migratory potential, curtails differentiation, impedes DNA reparative processes, and is intricately woven into the mitochondrial metabolism governing the cell cycle. Consequently, its role as an oncogene in specific neoplasms is undeniable [15]. CCND1, perceived as a mitotic cellular sentinel, can, when dysregulated, plunge the cell into cycles of disarray and dysfunction [16]. Given its frequent amplification in malignancies [17], CCND1 remains a cynosure in oncologic pursuits [18]. Dickkopf-related protein 1 (Dkk1), a protein consisting of 266 amino acids, manifests predominantly within mature tissues, including bone, placenta, prostate, spleen, and colon. It operates as a formidable adversary to the Wnt signaling cascade. DKK1 obstructs the intricate interplay between Wnt, FZD, and LRP6, culminating in the degradation of β-catenin and the subsequent dormancy of the β-catenin/T-cell-specific factor (TCF) transcriptional ensemble, thereby attenuating genes steered by T-cell factors. Clinical evaluations across a spectrum of malignancies have detected amplified concentrations of this Wnt antagonist, Dickkopf-1(DKK1), within patient sera and neoplasms, frequently serving as harbingers of an unfavorable prognosis. DKK1 exhibits prowess in modulating immune cellular dynamics and curbing the tumoral immunosuppressive milieu. Its involvement in T-cell differentiation and fostering tumor subterfuge from immune scrutiny, primarily through the proliferation of MDSCs, has thrust DKK1 into the spotlight as a prospective linchpin in cancer immunotherapy [19]. IIt acts as a catalyst in the tumorigenesis of pancreatic, esophageal, and hepatocellular carcinomas, stands as a prognostic marker in breast, gastric, and colorectal malignancies, and spurs prostate neoplastic cell growth and migration [20–26]. Additionally, DKK1 orchestrates the accrual and function of MDSCs in malignancies, is ubiquitously acknowledged as a potent tumor-affiliated antigen in multiple myeloma, and has been anointed as an innovative immunotherapy target for myeloma [27–29]. Contrarily, its role in mitigating breast neoplastic cell movement and infiltration is achieved through suppression of the β-catenin/MMP7 signaling conduit [30]. Ephrins serve as the ligands for the Eph receptor tyrosine kinase subset, with EFNB2 donning the dual hats of a receptor ligand and a signaling receptor. Its omnipresence within tumoral vasculature, coupled with its role in catalyzing tumor angiogenesis and neovascularization by anchoring vascular endothelial forerunners to neoplastic sites and its influence on lymphangiogenesis, underscores its significance. EFNB2 gene expression, fostering cellular proliferation, migration, and invasive tendencies particularly in pancreatic ductal adenocarcinoma, emerges as both a biological compass and a prognostic touchstone in a plethora of malignancies. Its expression intensity resonates with the malignancy’s severity [31–34]. Eph signaling’s intricate nexus with tumoral migration, growth, angiogenesis, apoptosis, and metastasis heralds its prospective therapeutic potential [35]. ITGA5, an affiliate of the integrin chain consortium, is an ever-present adhesive entity. These cellular sentinels are pivotal in adhesive dynamics and intracellular communication. They sculpt neovascularization patterns and wield influence over neoplastic growth, invasion, and metastatic spread via conduits like FAK and PI3K [36]. Its association spans from hepatocellular carcinomas to gliomas, including mesenchymal shifts in tongue squamous cell carcinomas [37–39]. Its prognostic worth extends to non-small cell lung neoplasms and pediatric acute myeloid leukemia [40, 41]. Augmenting this, curbing ITGA5 has been shown to bolster certain chemotherapeutic regimens’ efficacy [42]. Amphiregulin (AREG), a ligand specific to the epidermal growth factor receptor (EGFR), plays a pivotal role in the intricate orchestration of juxtacrine signaling amongst neighboring cells. In a distinct capacity, AREG is excreted, functioning as an autocrine or paracrine mediator. Its gene expression and subsequent discharge are precipitated by a myriad of agents, encompassing inflammatory lipids, cytokines, hormones, growth factors, and foreign substances [43]. By binding to EGFR, AREG triggers a slew of salient intracellular signaling pathways that delineate cellular viability, proliferation, and dynamism [44]. Exogenously secreted AREG, upon interfacing with EGFR, instigates a self-propagating feedback loop that enhances AREG transcription. Multiple intracellular conduits, such as MAPK, PI3K/Akt, STAT, and mTOR, are activated in the wake of AREG interaction, serving as the fulcrum for AREG/EGFR-directed cellular operations [45]. AREG has also been associated with oxidative stress and ferroptosis pathways, and HIF2-α regulates AREG, which are consistent with the results in the literature on AREG-induced transcriptional pathways under hypoxic conditions [46]. Studies have also shown that HIF-2α can enhance oxidative death in colon cancer cells through ferroptosis activators and DMF [47]. Areg is involved in immune regulation; can be an autocrine factor for tissue Treg, Treg expresses EGF receptors; and enhances Treg function [48]. It also enhances the differentiation of Th9 cells [49]. Mast cell-derived AREG enhances the immunosuppressive capacity of regulatory T (Treg) cells, except for AREG expressed by cancer cells in specific cases, which produce acquired resistance to anti-EGFR therapies (e.g., cetuximab), and sensitivity to cetuximab depends on high expression of both EREG and AREG [50, 51]. AREG is also associated with hepatocellular carcinoma, cholangiocarcinoma, pancreatic cancer, lung cancer, and breast cancer [52–59]. Studies have shown that AREG can promote multiple cancer models in the invasion and in regulating tumorigenesis [60, 61] and that targeted stromal-derived AREG can eliminate the AREG generated by stromal and cancer cells at the TME ecological site [50] and can be used in combination with anti-PD-1 antibodies [62].

EPHX3, an adept epoxide hydrolase, proficiently metabolizes volatile xenobiotic epoxides and modulates endogenous epoxides integral to cellular signaling. With pronounced expression in the proximal digestive tract, bone marrow, lymphatic structures, and dermal layers, EPHX3 emerges as a paramount arbiter of tumorigenesis across 13 malignancies. In the context of HNSCC, it not only epitomizes a prognostic indicator but also manifests its antineoplastic prowess by curbing tumor immune checkpoint articulation and immune cellular permeation [63]. EPHX3 hypermethylation may contribute to the development of OSCC and is associated with adenoid cystic carcinogenesis and progression [64]. Chromophobe granule B (CHGB) is one of the two major soluble proteins in the chromophobe granules of the adrenal medulla [65]. CHGB, instrumental in immune modulation, exhibits deviant gene expression across myriad tumor varieties, with its augmented expression being intrinsically linked to metastatic events. It may be a prognostic marker in neuroendocrine tumors, correlates with survival in colon cancer, correlates with malignant behavior in pheochromocytomas and abdominal paragangliomas, and correlates with poor prognosis in several squamous cell carcinomas (SCCs), namely HNSCC [66–69]. P4HA1 expression, catalyzed by HIF-1 under hypoxic conditions, orchestrates various tumorigenic pathways, including EMT, angiogenesis, invasion, inflammation, and notably, the glycolytic process. P4HA1 has an important role in the HIF-1 signaling pathway [70] and is associated with various tumors, namely HNSCC, breast cancer, colorectal cancer, and B-cell lymphoma [71–75]. JCHAIN is a polypeptide chain specific to polymeric immunoglobulins (Igs) and is thought to be responsible for linking monomeric subunits into a polymeric form [76]. It is required for the multimerization of IgM and IgA, a small polypeptide required for the transport of these Ig classes across the mucosal epithelium in a multi-Ig receptor-mediated process [77] associated with various cancers, namely ovarian, gastric, and breast cancers [78–80]. JCHAIN is linked to both inherent and adaptive resistance to radiation therapy in nasopharyngeal carcinoma patients, influencing their prognostic outcomes [81].

We established three molecular subtypes, C1-C3, based on prognostically significant differential mitochondria-related genes, which were associated with cell cycle-related and tumorigenesis-related pathways, with cell cycle-related pathways being inhibited in C1 and cell cycle-related and tumorigenesis-related pathways being activated in C3. The differential genes of the subtypes were further analyzed by one-way Cox analysis and multifactor stepwise regression analysis. In the final analysis, nine pivotal genes were discerned as instrumental in influencing prognosis. Notably, DKK1, EFNB2, AREG, CHGB, P4HA1, and CCND1 manifested as risk determinants, while ITGA5, EPHX3, and JCHAIN emerged as protective indicators. The RiskScore affirmed the model’s robustness, evident from the substantial area beneath the AUC curve, delineating marked disparities between high-risk and low-risk cohorts. The mRNA expression corroborated in clinical specimens mirrored largely congruent patterns; predominantly, there was a pronounced diminution in tumorous tissues, juxtaposed with an amplification in normal samples. The RiskScore showed a significant negative correlation with most immune cell scores. The RiskScore was positively correlated with glycolysis, angiogenesis, hypoxia, and tumor-related pathways (WNT, TFGβ, EMT) and further influenced immune regulation. These phenomena were verified using the GSE41613 and GSE65858 datasets.

In summation, we devised a risk paradigm informed by nine mitochondria-centric genes, synergizing the RiskScore with clinicopathological nuances to elevate the precision of our prognostic framework and survival foresight, which boasted notable predictive acuity. Moreover, we scrutinized variances within the immune milieu across risk categories, discerning that the RiskScore harbored a marked inverse association with the majority of immune cell evaluations. We further gauged the responsiveness of distinct subgroups to traditional chemotherapy regimens. This model stands as a salient tool in HNC, facilitating therapeutic guidance and tailoring patient-centric interventions.

## MATERIALS AND METHODS

### Data collection and processing

Utilizing the TCGA GDC API, we procured RNA-seq data from TCGA-HNSC. Post meticulous selection, we incorporated 499 primary tumor specimens and 44 normative samples. From the GEO repository, we garnered expression metrics for GSE41613, leading to the inclusion of 97 distinguished samples. Similarly, for GSE65858, post-evaluation, 270 samples were integrated.

### Source of mitochondria-related genes

The mitochondria-associated genes were obtained from the literature [82]. Mitochondria-associated genes encode mitochondria-localized proteins, namely all proteins in the mitochondrial membrane, matrix, cristae, and mitochondria-associated endoplasmic reticulum membrane. On the basis of subcellular localization, all genes were obtained from the Molecular Signature Database (MSigDB) database (http://software.broadinstitute.org/gsea/msigdb) for a set of 23 mitochondria-associated cellular component genes; 1576 genes were identified as mitochondria-associated genes (Supplementary Table 9).

### Data preprocessing

From TCGA, RNA-seq data underwent a meticulous four-phase refinement:

1. Exclusion of specimens lacking clinical follow-up data.
2. Preservation of samples boasting survival durations exceeding 0 days.
3. Elimination of samples devoid of status.
4. Retain coding protein genes.

For the GEO dataset, the subsequent procedures were executed:

Upon accessing the pertinent microarray platform’s annotation details, probes were aligned with genes in accordance with this annotated data. Probes correlating with multiple genes were excised. Conversely, when several probes corresponded to a singular gene, their mean expression served as the definitive gene expression value.

### Molecular subtyping of mitochondria-related genes

We constructed consistency matrices by using ConsensusClusterPlus for the clustering samples [83]. We obtained the molecular subtypes of the model by using mitochondria-related gene expression data with a significant prognosis. Utilizing the “km” algorithm with “Euclidean” as the distance metric, we executed five hundred bootstraps, each encompassing 80% of the patients from the training set. The cluster range was established between 2 and 10. The optimal classification emerged from evaluating the consistency matrix and the CDF, thereby discerning the molecular subtypes of the specimen.

### Risk model

1. Identify the differentially expressed genes between subtypes via the molecular subtypes identified previously (|log2FC|>1 and FDR <0.05).
2. Select genes with a significant prognosis (*P* < 0.001).
3. Further reduce the number of genes by using multifactor stepwise regression to obtain the prognostically significant genes associated with the mitochondrial phenotype.
4. Conduct risk modeling. The risk score for each patient was determined using the equation: RiskScore = Σβi × Expi. In this equation, ‘I’ denotes the expression level of genes associated with mitochondrial phenotype prognostic features, and β’ represents the pertinent gene’s Cox regression coefficient. Patients were stratified into two categories—high risk and low risk based on the median risk score. Survival curves were crafted using the Kaplan-Meier approach, with the log-rank test assessing the significance of observed disparities.

### GSEA

To investigate the pathways of different biological processes in different molecular subtypes, we used “GSEA” for pathway analysis: we performed gene set enrichment analysis using candidate gene sets from the HALLMARK database.

### Calculation of TME cell invasion abundance

The CIBERSORT algorithm ascertained the proportional abundances of 22 immune cells within the tumor tissue. Immune infiltration was gauged via the ESTIMATE software, while the MCP Count function assessed the metrics of 10 immune cells. Conversely, the TIMER function evaluated the metrics of six distinct immune cells.

### Prediction of responsiveness to immunotherapy

The TIDE algorithm, a computational stratagem forecasting ICB responsiveness via gene expression profiling, authenticated the impact of IMS on predicting clinical receptivity to ICIs [84]. This algorithm scrutinized three cellular entities impeding T-cell penetration in tumors: the M2 variant of CAF, MDSCs, and TAMs. Moreover, it appraised two distinct tumor immune evasion methodologies: the compromised efficacy of tumor-infiltrating CTLs and the counteraction of CTLs due to immunosuppressive elements.

### qRT-PCR

TRIzol facilitated the extraction of total RNA from pristine human HNSCC specimens and adjacent tissues, subsequently undergoing reverse transcription to cDNA. For the scope of this investigation, the patient consented to the surgical utilization of human tissue at the Quanzhou First Hospital, affiliated with Fujian Medical University, spanning October 2021 to November 2022. This endeavor received the endorsement of the Ethics Committee of the said institution, affirming the projected blueprint Quantitative real-time PCR, standardized to actin, was executed on the ABI 7900 apparatus employing the SYBR Green RT-PCR assay. The following primers were used for PCR: AREG: 5′-CTGTCGCTCTTGATACTCG-3′ (sense), 5′-CAGAAAATGGTTCACGCT-3′ (antisense); DKK1: 5′-AACTGGGAGAAGATGGCT-3′ (sense), 5′-TCCTGGGGTGAAAGTATG-3′ (antisense); and EFNB2: 5′-ACATTCGGGGAACAACAT-3′ (sense), 5′-TTCAGCAAGAGGACCACC-3′ (antisense).

### Statistical analysis

Analyses were executed with GraphPad Prism 5 and R (version 3.6.3), representing data as mean ± SD. The Student’s *t*-test assessed differential expression across tissues. The Cox function in R facilitated the Univariate Cox analysis, elucidating associations with HNSCC prognosis. Prognostic evaluations employed Kaplan-Meier survival curves, with the log-rank test discerning the significance of disparities. A threshold of *P* < 0.05 demarcated statistical significance.

### Data availability

The data used to support the findings of this study have been included in this article.

## Supplementary Materials

Supplementary Table 1

Supplementary Table 2

Supplementary Table 3

Supplementary Table 4

Supplementary Table 5

Supplementary Table 6

Supplementary Table 7

Supplementary Table 8

Supplementary Table 9
